# Improved n-butanol production via co-expression of membrane-targeted tilapia metallothionein and the clostridial metabolic pathway in *Escherichia coli*

**DOI:** 10.1186/s12896-017-0356-3

**Published:** 2017-04-11

**Authors:** Wei-Chih Chin, Kuo-Hsing Lin, Chun-Chi Liu, Kenji Tsuge, Chieh-Chen Huang

**Affiliations:** 1 Department of Life Sciences, National Chung Hsing University, Taichung, Taiwan; 2 Center of Cold Chain Logistics Certification, College of Management, National Kaohsiung First University of Science and Technology, Kaohsiung, Taiwan; 3 Institute of Genomics and Bioinformatics, National Chung Hsing University, Taichung, 402 Taiwan; 4 Graduate School of Science, Technology and Innovation, Kobe University, Kobe, Japan

**Keywords:** Tilapia metallothionein, OmpC, n-butanol, *E. coli*, Oxidative stress, Transcriptomic analysis

## Abstract

**Background:**

N-Butanol has favorable characteristics for use as either an alternative fuel or platform chemical. Bio-based n-butanol production using microbes is an emerging technology that requires further development. Although bio-industrial microbes such as *Escherichia coli* have been engineered to produce n-butanol, reactive oxygen species (ROS)-mediated toxicity may limit productivity. Previously, we show that outer-membrane-targeted tilapia metallothionein (OmpC-TMT) is more effective as an ROS scavenger than human and mouse metallothioneins to reduce oxidative stress in the host cell.

**Results:**

The host strain (BUT1-DE) containing the clostridial n-butanol pathway displayed a decreased growth rate and limited n-butanol productivity, likely due to ROS accumulation. The clostridial n-butanol pathway was co-engineered with inducible OmpC-TMT in *E. coli* (BUT3-DE) for simultaneous ROS removal, and its effect on n-butanol productivity was examined. The ROS scavenging ability of cells overexpressing OmpC-TMT was examined and showed an approximately twofold increase in capacity. The modified strain improved n-butanol productivity to 320 mg/L, whereas the control strain produced only 95.1 mg/L. Transcriptomic analysis revealed three major KEGG pathways that were significantly differentially expressed in the BUT3-DE strain compared with their expression in the BUT1-DE strain, including genes involved in oxidative phosphorylation, fructose and mannose metabolism and glycolysis/gluconeogenesis.

**Conclusions:**

These results indicate that OmpC-TMT can increase n-butanol production by scavenging ROS. The transcriptomic analysis suggested that n-butanol causes quinone malfunction, resulting in oxidative-phosphorylation-related *nuo operon* downregulation, which would diminish the ability to convert NADH to NAD^+^ and generate proton motive force. However, fructose and mannose metabolism-related genes (*fucA*, *srlE* and *srlA*) were upregulated, and glycolysis/gluconeogenesis-related genes (*pfkB*, *pgm*) were downregulated, which further assisted in regulating NADH/NAD^+^ redox and preventing additional ATP depletion. These results indicated that more NADH and ATP were required in the n-butanol synthetic pathway. Our study demonstrates a potential approach to increase the robustness of microorganisms and the production of toxic chemicals through the ability to reduce oxidative stress.

**Electronic supplementary material:**

The online version of this article (doi:10.1186/s12896-017-0356-3) contains supplementary material, which is available to authorized users.

## Background

n-Butanol has many advantages over ethanol, including a higher energy density due to two extra carbons, and can be used in gasoline engines without modification. n-Butanol is less hygroscopic and volatile than ethanol and has been recently regarded as a more viable transportation biofuel than ethanol [1–3]. n-Butanol is also a versatile platform chemical that can be produced from a variety of biomass sources. In industry, many important chemicals derived from n-butanol are used extensively as solvents or as intermediates in the production of acrylates, n-butyl acetate, esters, and glycol ethers [4].

Several industrial microbes, including *Escherichia coli*, have been engineered to produce n-butanol. In the initial stage of engineering *E. coli* for n-butanol production, the whole n-butanol pathway from *Clostridium* was transferred to *E. coli*, including the *thil*, *hbd*, *crt*, *bcd*, *etfA*, *etfB*, and *adhe* (or *adhel*) genes, catalyzing the six-step conversion of two molecules of acetyl-CoA into one molecule of n-butanol. However, when the clostridial n-butanol pathway was first transferred to *E. coli* using plasmids in 2007, the engineered strain produced less than 1 g/L n-butanol (vs. a clostridial n-butanol titer of 10–20 g/L) [5, 6]. The results indicated that engineering efficient n-butanol-producing *E. coli* is not as simple as expressing several clostridial n-butanol pathway genes. Over the following years, the best heterologous n-butanol-producing strains derived from *E. coli* were able to produce 14–15 g/L n-butanol [7, 8], providing industrial advantages compared to clostridial strains [9]. However, the toxicity of n-butanol to both natural producers and engineered hosts increases with accumulation [10, 11]. This toxicity complicates the economically efficient production of large titers of n-butanol; therefore, high-titer n-butanol (30 g/L) production currently relies on in situ product removal by continuous gas stripping [8].

Furthermore, although *E. coli* can convert sugars into n-butanol at relatively high levels, the cells cannot tolerate 2% (v/v) n-butanol [11] and produce n-butanol at insufficient levels. Considering the relationship between n-butanol tolerance and n-butanol production in *Clostridial* strains [12, 13], the toxicity of n-butanol to *E. coli* can be considered a production bottleneck. In fact, product toxicity is a widespread problem in the production of biorenewables. Therefore, the development of a stress-tolerant host strain for the bio-production of titer-dependent toxic chemicals is important.

To understand the effect of n-butanol toxicity on the host, cell-wide studies have been conducted to obtain a global view of the n-butanol stress-response at the transcript, protein, and metabolite levels. Transcriptomic analysis in *Clostridium acetobutylicum* indicated that the primary response involved the accumulation of transcripts encoding chaperones, proteases, and other heat shock-related proteins [14].

In *E. coli*, several transcriptional analyses have been performed to investigate the stress caused by alcohols, including ethanol, n-butanol, and isobutanol [15–17]. Additionally, observations from fluorescent dye-staining indicated a large increase in reactive oxygen species (ROS) during n-butanol stress [17]. Increased oxidative stress is a common cellular response to extracellular xenobiotics, which may mediate macromolecular damage. These free radicals can directly attack the membrane via lipid peroxidation or cause DNA mutations, protein misfolding and fragmentation, and apoptosis [18–20].

To decrease ROS-induced oxidative damage, microorganisms synthesize many antioxidant enzymes, including catalases, superoxide dismutases and glutathione peroxidase [21, 22]. Recently, metallothioneins (MTs), beneficial antioxidant enzymes that occur widely in mammals, plants and fungi, have been identified [23]. MTs are heat-stable, low-molecular-weight and cysteine-rich intracellular proteins [23–25]. MTs also act as a defense system against oxidative stress through their ROS scavenging ability [26]. Furthermore, purified tilapia MT (TMT) has been shown to outperform glutathione (GSH) in scavenging both 2-diphenyl-1-picrylhydrazyl (DPPH^●^) and 2,2-azinobis (3-ethylbenzothiazoline- 6-sulfonic acid) diammonium salt (ABTS^●+^) [27]. To reduce oxidative stress in the host cell, we previously engineered MTs into *E. coli* hosts for both cytosolic and outer-membrane-targeted (osmoregulatory membrane protein, OmpC, fused) expression. The ability of these engineered *E. coli* to scavenge intracellular or extracellular ROS was examined, and OmpC-fused TMT performed the best, growing in medium containing 1.5% n-butanol. n-Butanol tolerance was increased through the scavenging of intracellular and extracellular free radicals, and the fusion protein still contributed to osmotic tolerance via either glycine betaine or glucose uptake [28].

Intracellular ROS increase levels in *E. coli* after exposure to n-butanol [17]. We therefore considered whether introducing OmpC-fused TMT into butanologenic *E. coli* would improve n-butanol tolerance and productivity. In the present study, we engineered the *C. acetobutylicum* n-butanol synthetic pathway into *E. coli*, along with OmpC-TMT co-expression to increase n-butanol tolerance. A synthetic biological technical platform (Ordered Gene Assembly in *Bacillus subtilis* – OGAB method) was employed for the genetic manipulation of the n-butanol production and tolerance gene clusters [29]. Our results demonstrate that co-expressing OmpC-fused TMT in a butanologenic *E. coli* strain enhances n-butanol production.

## Results and discussion

### Construction of butanologenic *E. coli* strains

**Fig. 1:**
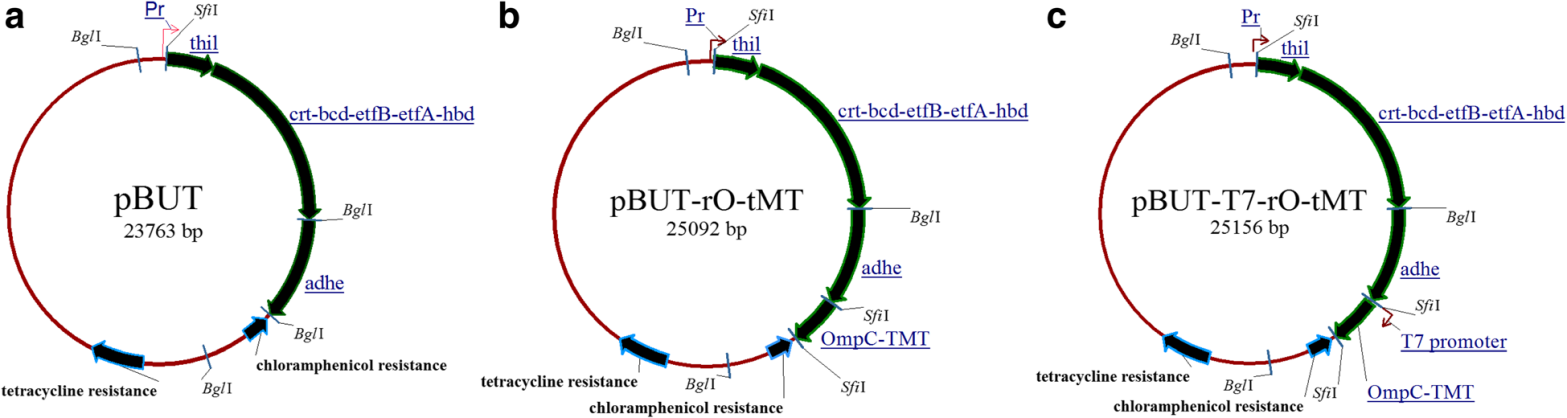
Structure of the plasmids pBUT, pBUT-rO-tMT and pBUT-T7-rO-tMT. Three recombinant plasmids were derived from the expression vector pGETS118 [29, 74], which contained the Pr promoter. To assemble the butanol synthesis pathway, target gene(s) *thil* (1.2 kb), *crt*-*bcd*-*etfB*-*etfA*-*hbd* cluster (4.8 kb), *adhe* (2.8 kb) and *rO*-*tMT* (1.3 kb) or *T7*-*rO*-*tMT* (1.4 kb) were inserted under the Pr promoter, resulting in pBUT (**a**), pBUT-rO-tMT (**b**), and pBUT-T7-rO-tMT (**c**), respectively. All the plasmids include two antibiotic resistances (Tc^r^ : tetracycline resistance; CAT^r^ : chloramphenicol resistance)

To establish butanologenic *E. coli* strains, we transferred only an essential set of genes (*thil*, *crt*, *bcd*, *etfAB*, *hbd*, *adhe*) for n-butanol production [5, 6]. In addition, our previous results indicated that the expression of the *ompC-tmt* gene enhances the tolerance for n-butanol through osmotic tolerance and extracellular radical scavenging capacity [28]. These genes were cloned and constructed in the plasmid pGETS118 using the OGAB method [29]. The plasmids pBUT, containing *Pr*-*thil*-*crt*-*bcd*-*etfAB*-*hbd*-*adhe*, pBUT-rO-tMT, containing *Pr*-*thil*-*crt*-*bcd*-*etfAB*-*hbd*-*adhe*-*rbs*-*ompC*-*tmt*, and pBUT-T7-rO-tMT, with *Pr*-*thil*-*crt*-*bcd*-*etfAB*-*hbd*-*adhe*-T7 promoter-*rbs*-*ompC*-*tmt*, were constructed. The constructed plasmids pBUT, pBUT-rO-tMT and pBUT-T7-rO-tMT (Fig. 1) were used to transform *E. coli* JM109 and *E. coli* JM109 (DE3) to obtain the butanologenic *E. coli* strains BUT1, BUT2, BUT3, BUT1-DE, BUT2-DE and BUT3-DE. Finally, pGETS118 was used to transform *E. coli* JM109 and *E. coli* JM109 (DE3) to obtain the control strains PGETS118 and PGETS118-DE (Table 1). Cell growth and n-butanol production were then investigated.

**Table 1:** Strains and plasmids used in this study

Strains and plasmids	Genotype and description^a^	Reference or source
*E. coli* strains		
pET-OmTmt	*E. coli* BL21/PET30a, T7 promoter, f1 origin; *ompC-tmt*, Km^r^	[27]
JM109	*endA1, recA1, gyrA96, thi, hsdR17 (rk–, mk+), relA1, supE44, Δ(lac-proAB), [F’ traD36, proAB, laqIqZΔM15].*	Promega
PGETS118	*E. coli* JM109/pGETS118, CAT^r^	[75]
BUT1	*E. coli* JM109/pBUT, CAT^r^	This work
BUT2	*E. coli* JM109/pBUT-rO-tMT, CAT^r^	This work
BUT3	*E. coli* JM109/pBUT-T7-rO-tMT, CAT^r^	This work
JM109 (DE3)	JM109 *+ λ(DE3),*Contains an IPTG-inducible gene for T7 RNA polymerase	Promega
PGETS118-DE	*E. coli* JM109 (DE3)/pGETS118, CAT^r^	This work
BUT1-DE	*E. coli* JM109 (DE3)/pBUT, CAT^r^	This work
BUT2-DE	*E. coli* JM109 (DE3)/pBUT-rO-tMT, CAT^r^	This work
BUT3-DE	*E. coli* JM109 (DE3)/pBUT-T7-rO-tMT, CAT^r^	This work
Plasmids		
pGETS118	pGETS118SfiI-Pr, Tc^r^, CAT^r^	This work
pBUT	pGETS118::*thil*-*crt*-*bcd*-*etfAB*-*hbd*-*adhe*, Tc^r^, CAT^r^	This work
pBUT-rO-tMT	pBUT::rbs-*ompC*-*tmt*, Tc^r^, CAT^r^	This work
pBUT-T7-rO-tMT	pBUT::T7 promoter-*ompC*-*tmt*, Tc^r^, CAT^r^	This work

^a^CAT^r^, chloramphenicol resistance; Km^r^, kanamycin resistant

Tc^r^, tetracycline resistance; Pr, Pr promoter; cI, cI cassette

### Growth profile and n-butanol production

Recently, many groups have reported successful n-butanol production in *E. coli* [5, 6, 8]; a similar approach was previously applied to n-butanol production in *E. coli* carrying the CoA-dependent synthetic pathway, yielding final n-butanol titers of 552 mg/L to 1.2 g/L [5, 6]. In addition, Shen et al. [8] further improved the strain by using NADH- and acetyl-CoA-driving forces; the best strain in their study showed high-titer production of n-butanol through in situ product removal. The production of n-butanol has been confirmed to affect host growth.

**Fig. 2:**
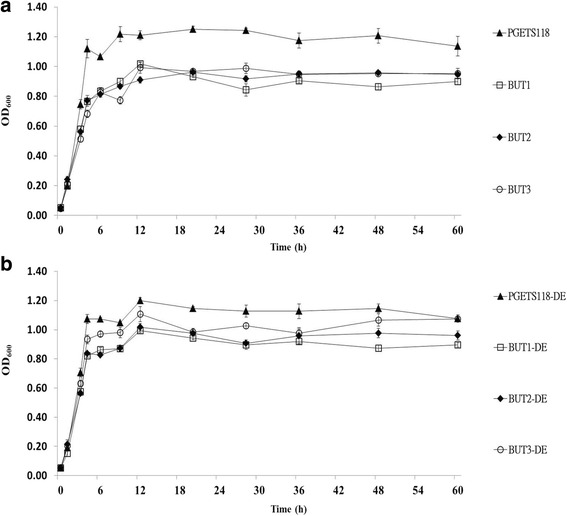
Growth of engineered *E. coli* strains with various vectors. The engineered *E. coli* were cultured in PYG medium with different expression hosts (**a**.) JM109 and (**b**.) JM109 (DE3). All strains were grown under anaerobic conditions at 37 °C for 60 h. The values and error bars are based on three replicate experiments

**Fig. 3:**
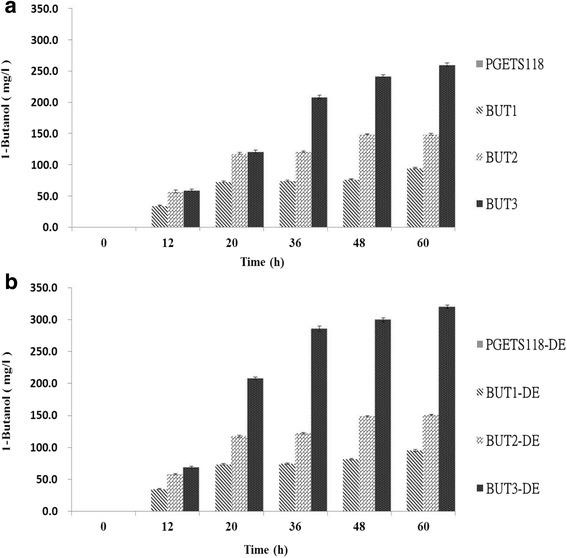
Comparison of n-butanol production in *E. coli* containing various vectors. The engineered *E. coli* were cultured in PYG medium with different expression hosts (**a**.) JM109 and (**b**.) JM109 (DE3). All strains were grown under anaerobic conditions at 37 °C for 60 h. The values and error bars are based on three replicate experiments

Compared with the control strains PGETS118 and PGETS118-DE, both recombinant strains BUT1 and BUT1-DE displayed increased n-butanol titer (both reached approximately 73 mg/L) after 20 h. Furthermore, 17-21% reductions in the growth of both strains in PYG medium were observed (Figs. 2 and 3). In addition, after 60 h of incubation, maximum n-butanol concentrations of 94.6 mg/L and 95.1 mg/L were recorded in BUT1 and BUT1-DE cultures, respectively. This result shows that cells at an OD_600_ value of 1 can convert 1 g of glucose to 1.75 mg/L of n-butanol in PYG medium, similar to the rate reported by Inui et al. [6] (1.5 mg/L n-butanol/1 g glucose). In the case of PGETS118 and PGETS118-DE, which were monitored as controls, no n-butanol was detected.

### Improved tolerance and n-butanol production

A clostridial strain overexpressing GSH was reported to exhibit improved tolerance and increased n-butanol productivity [30]. Boyarskiy et al. [31] reported increased n-butanol tolerance and production (approximately 35% increases) through transcriptional feedback regulation of efflux protein expression.

In the present study, we attempted to improve the n-butanol tolerance of butanologenic *E. coli* to further enhance n-butanol production by expressing OmpC-TMT. The concentrations of n-butanol found to be inhibitory to *E. coli* in n-butanol production strains (Fig. 2) were consistent with the trends noted in the literature for alcohol toxicity in *E. coli* [17]. Hence, we examined the normal expression of OmpC-TMT (BUT2, BUT3) in butanologenic *E. coli* strains, and the results showed that cell growth was still affected by n-butanol. This result is similar to that obtained for the BUT1 strain (Fig. 2a).

However, the BUT2 and BUT3 strains showed similar n-butanol production (118 mg/L and 121 mg/L) after 20 h of incubation (Fig. 3a). After incubation for 60 h, the butanologenic *E. coli* strains BUT2 and BUT3 showed 1.6-fold and 2.7-fold higher n-butanol production, respectively, than BUT1 (94.6 mg/L). Notably, BUT3-DE achieved a 1.7-fold increase in n-butanol production compared to that achieved by BUT2-DE after 20 h of incubation (Fig. 3b). Furthermore, cell growth in the OmpC-TMT-overexpressing butanologenic *E. coli* (BUT3-DE) was nearly the same as in the control strain (PGETS118-DE) after 60 h of incubation (Fig. 2b). More importantly, n-butanol production increased nearly 3.4-fold compared with that of BUT1-DE (320 mg/L) and showed a significantly improved conversion rate from glucose to n-butanol (4.97 mg/L n-butanol/1 g glucose) (Fig. 3b). These results suggested that OmpC-TMT overexpression rescued the growth of the butanologenic *E. coli* strains, improved host robustness and increased n-butanol productivity. Our results are consistent with previous reports [28, 30, 31].

In addition, many genes were related to organic solvent tolerance in the *E. coli* strain, for example, mutant cyclic AMP receptor protein (CRP) [32], overexpression of *groESL* [33–35], mutant Δ*lon* (cell envelope-related gene) [36] and control membrane-related functions (overexpression of ATF, *fabD*, *feoA* and *srpABC*) [37]. Moreover, Rutherford et al. [17] reported that n-butanol stress-response genes are also involved in many stress responses, such as oxidative stress (*sodA*, *sodC* and *yqhD*), heat shock and cell envelope stress (*rpoE*, *clpB*, *htpG*, *cpxR* and *cpxP*). Recently, the tolerance mechanisms of several critical genes have been elucidated. Among them, the downregulated genes *yghW* and *yibT* were shown to improve n-butanol tolerance due to their regulatory roles in membrane fatty acid composition, while the upregulated genes *gcl* and *glcF* improved cell growth and metabolism by replenishing TCA cycle metabolic intermediates [38]. However, these results reflect the addition of n-butanol to the medium rather than its production by *E. coli* (Table 2).

**Table 2:** Engineering strategies to improve butanol tolerance and production in *E. coli*

Butanol tolerance				Butanol production		
	Strategy	Tolerance (v/v)	Reference	Strategy	Production (mg/l)	Reference
1	Overexpression of *ompC-tmt*	From 1.50 to 2.00%	[28]	*thil*, *crt*, *bcd*, *etfA*, *etfB*, *hbd*, *adhe*, *ompC*-*tmt*	320 mg/l	This work
2	N.D.	N.D.	N.D.	*atoB*, *crt*, *bcd*, *etfAB*, *hbd*, *adhe*, *ΔadhE*, *ΔldhA*, *ΔfrdBC*, *Δfnr*, *Δpta*	552 mg/l	[5]
3	N.D.	N.D.	N.D.	*thil*, *hbd*, *crt*, *bcd*, *etfA*, *etfB*, *adhe*, high cell density	1,200 mg/l	[6]
4	Overexpression of ATF, *fabD*, *feoA* and *srpABC*	From 1.50 to 2.00%	[38]	N.D.	N.D.	N.D.
5	double disruptions of proV and marR Mutation of *lon*	From 1.00 to 2.00%	[37]	N.D.	N.D.	N.D.
6	Overexpression of *groESL*	From 0.75 to 1.00%	[34]	N.D.	N.D.	N.D.
7	Mutation of cyclic AMP receptor protein (CRP)	From 0.80 to 1.20%	[33]	N.D.	N.D.	N.D.

*N.D.* no data

Aside from n-butanol, carboxylic acids are attractive biorenewable chemicals, such as fatty acid and aromatic carboxylic acids [39–41]. However, product toxicity is frequently encountered in metabolic engineering, and the toxicity of these carboxylic acids to the microbial biocatalyst appears to limit biocatalyst performance. Strong evidence suggests that membrane damage is the main mechanism of toxicity [42, 43], and previous metabolic engineering efforts successfully increased membrane integrity by modulating membrane composition to alter free fatty acid tolerance in *E. coli* [44–46].

### Free radical scavenging ability and membrane integrity test

Since the cellular membrane is a vital factor that allows cells to acclimate to external stresses and is also one of the components that is strongly affected by organic solvents [47], many studies have proposed that the plasma membrane is the most affected target of organic solvents and plays a significant role in adapting to stress. Additionally, the length of the carbon backbone of the organic solvent can alter the toxicity mechanism; increasing the hydrophobicity of the solvent increases the level of toxicity [48]. Long- and short-chain alcohols cause stress during biofuel production by altering membrane fluidity (also known as Overton’s Rule). Ethanol and n-butanol, respectively, decrease and increase membrane fluidity [47, 49, 50].

Toxicity that causes membrane damage is likely a key limitation for butanologenic microbes to produce butanol, suggesting that our engineering strategy of expressing OmpC-TMT is effective in protecting membrane integrity and scavenging free radicals to improve n-butanol tolerance and production in butanologenic *E. coli* strains.

**Fig. 4:**
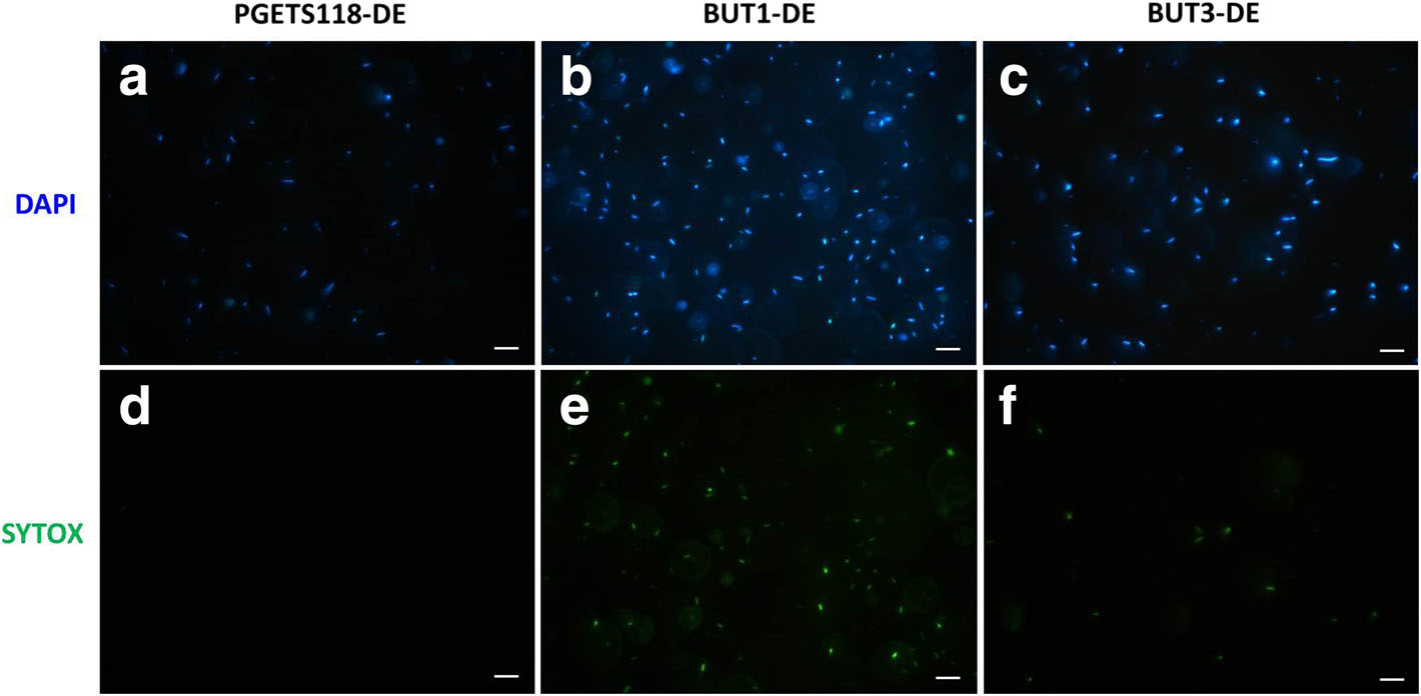
Fluorescence microscopy of DAPI- and SYTOX *Green*-labeled bacteria. Three kinds of the engineered *E. coli* strains (PGETS118-DE, BUT1-DE and BUT3-DE) were cultured in PYG medium and incubated at 37 °C for 48 h. All bacteria were stained with both DAPI and SYTOX *Green* nucleic acid stains. The staining patterns of *E. coli* labeled with 1 μg/ml DAPI and 5 μM SYTOX *Green* were compared via fluorescence microscopy. Viable bacteria or membrane-intact cells were stained and appear *blue* only (**a**, **d**). Nonviable bacteria or membrane-damaged cells are stained and appear *blue* and *green* (**b**, **e** and **c**, **f**). Scale bar = 5 μm. Measurements were obtained from three replicate experiments

To test whether OmpC-TMT increased membrane integrity during fermentation, bacteria were stained with DAPI and SYTOX Green nucleic acid stains after 48 h of incubation (Fig. 4). Viable bacteria or cells with membrane intact cells were stained and appeared blue only (a, d), whereas nonviable bacteria or cells with damaged membranes would appeared blue and green (b, e and c, f). The ratio of cell membrane damage was determined as the number of SYTOX stain-labeled bacteria cells/the number of DAPI stain-labeled bacteria cells and was 1.57% ± 0.33%, 48.59% ± 5.98%, and 19.69% ± 4.16%, for PGETS118-DE, BUT1-DE and BUT3-DE, respectively. The engineered *E. coli* strain BUT3-DE, expressing the outer-membrane fusion protein OmpC-TMT, exhibited better membrane integrity than that of *E. coli* strain BUT1-DE. These results clarified the relationship between membrane integrity and butanol resistance.

**Fig. 5:**
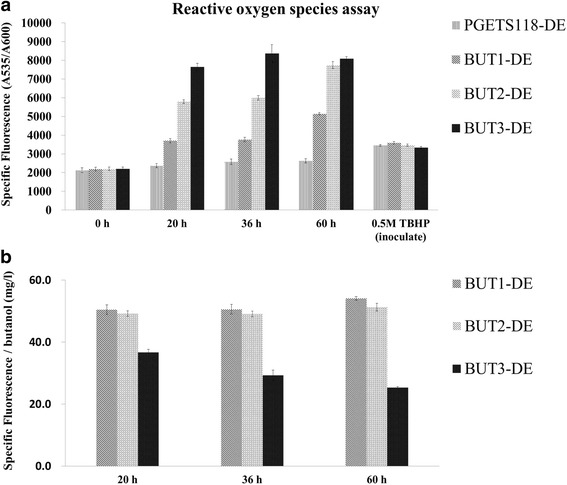
Quantitative assay of intracellular reactive oxygen species in different engineered *E. coli* strains. **a** The levels of free radicals in all strains. **b** The effect of n-butanol on each strain. The optical density at 600 nm and fluorescence at 535 nm of the engineered *E. coli* strains were measured for cells cultured in PYG medium for different times (0 h, 20 h, 36 h, 60 h) at 37 °C. “TBHP” represents the positive control in which strains were treated with 0.5 M tert-butyl hydroperoxide, which is known a stressor, after inoculation to produce intracellular H_2_O_2_. Measurements were obtained from three replicate experiments

MTs are antioxidants that scavenge radicals, and alcohols are known to cause oxidative stress in *E. coli* [51]. In the present study, we examined the ability of OmpC-TMT to scavenge free radicals when the host cells were producing n-butanol. We then analyzed the ROS content of the cells using 5(6)-carboxy-2′, 7′- dichlorodihydro-fluorescein diacetate (carboxy-H_2_DCFDA). During the first 60 h of incubation, the levels of free radicals in all strains increased with increasing concentration of n-butanol, as shown by the increasing levels of fluorescence (Fig. 5a). After fermentation for 20 h, BUT3-DE displayed 2-fold higher free radical content and 2.8-fold greater n-butanol production than BUT1-DE (Figs. 3b and 5a). In contrast, both PGETS118-DE and BUT1-DE had lower levels of radicals than the OmpC-TMT-expressing strains, because no or less n-butanol was produced. Moreover, we examined the capacity for OmpC-TMT to scavenge free radicals when the host cells produced n-butanol. As shown in Fig. 3b, BUT3-DE (320.24 mg/L) produced more n-butanol than BUT2-DE (151.03 mg/L). However, both BUT2-DE and BUT3-DE produced similarly large amounts of ROS after 60 h of incubation (Fig. 5a). The effect of n-butanol on each strain was defined as [specific fluorescence of each strain (A535/A600)/(n-butanol production by each strain)]. BUT3-DE showed approximately 2-fold higher free radical scavenging capacity (25.27) than BUT1-DE (54.07) and BUT2-DE (51.25) (Fig. 5b). Interestingly, this result is consistent with the result from the membrane integrity test. Therefore, OmpC-TMT overexpression enhanced n-butanol production by lowering the levels of free radicals and increasing membrane integrity.

### Expression profiles of n-butanol and tolerance genes in *E. coli*

As shown in Table 3, the relative expression of the four genetic regions (*thil*, *crt*-*bcd*-*etfB*-*etfA*-*hbd*, *adhe, ompC-tmt*) with respect to the *rrsA* gene in the two sample types (control and experimental) for three cases (1. BUT1-DE and BUT2-DE; 2. BUT1-DE and BUT3-DE; and 3. BUT2-DE and BUT3-DE) was determined using qPCR assays. In this study, differential expression was observed between the OmpC-tMT strains and the control. In case 1, expression of the four genetic regions was lower in the control (BUT1-DE). BUT1-DE expressed *ompC-tmt*, but we did not introduce this gene into the plasmid. This phenomenon was observed in another study in which an *E. coli* strain was observed to tolerate solvents through the upregulation of OmpC [38, 52]*.* Therefore, gene expression may be derived from the host. Nevertheless, BUT2-DE exhibited high free radical scavenging capacity and high n-butanol yield compared with BUT1-DE (Fig. 5b), indicating that lower oxidative stress and higher n-butanol production may result from TMT protein expression. In case 2 with BUT3-DE, the expression of n-butanol production genes was still lower than in the control (BUT1-DE), but the *ompC-tmt* gene showed high expression due to the T7 promoter. Hence, we examined the ability of BUT3-DE to scavenge free radicals, and the result showed BUT3-DE had the greatest ability to reduce oxidative stress and the best n-butanol production (Figs. 3b and 5a). Finally, in case 3, BUT3-DE expressed more n-butanol production genes than BUT2-DE. These results again suggest that OmpC-TMT overexpression improved the robustness of the host to achieve better solvent production by decreasing the production of ROS.

**Table 3:** Real-time RT-PCR analysis

Sample	Gene expression log2 fold change ^a^ [log2 (2^-ΔΔCT (Exp-Control)^)]			
Experimental/Control	*thil*	*crt-bcd-etfB-etfA-hbd*	*adhe*	*ompC-tmt*
BUT2-DE/BUT1-DE	-7.79 ± 0.02	-8.64 ± 0.08	-7.72 ± 0.06	-1.40 ± 0.02
BUT3-DE/BUT1-DE	-3.09 ± 0.12	-4.14 ± 0.69	-0.76 ± 0.10	2.84 ± 0.09
BUT3-DE/BUT2-DE	4.70 ± 0.11	4.50 ± 0.68	6.96 ± 0.16	4.24 ± 0.08

^a^ The values represent ratio of gene expression log2 fold change and are means ± standard deviations from three independent experiments

(Gene expression log2 fold change: >0, ie. Up regulation; ≒0, ie. No change; < 0, ie. Down regulation)

### Transcriptomic analysis in engineered *E. coli*

Understanding the membrane stress response to solvents and alcohols may facilitate the engineering of microorganisms for improved toxin tolerance. Therefore, the stress responses of organisms such as *E. coli* to ethanol exposure have been widely studied [53–55], and information from these studies has been successfully adapted to engineer improved ethanologenic hosts [56–59]. Although both strains appeared to grow into stationary phase after 36 h of incubation, meaningful increases (*P*-value < 0.01) in their butanol titers were still observed after 48 h (Fig. 3b, BUT1-DE: 74.49 ± 1.09 mg/l (36 h), 81.55 ± 1.15 mg/l (48 h) and 95.19 ± 1.62 mg/l (60 h); and BUT3-DE: 285.94 ± 4.10 mg/l (36 h), 299.81 ± 2.92 mg/l (48 h) and 320.24 ± 2.56 mg/l (60 h)). Meanwhile, as shown by the levels of fluorescence after 36 h and 60 h, ROS levels increased in BUT1-DE but decreased in BUT3-DE (Fig. 5a). Because BUT3-DE and BUT1-DE exhibited opposite changes in oxidative stress after 60 h (Fig. 5b), the effects of OmpC-TMT expression on butanologenic *E. coli* strains were explored via transcriptomic analysis using next-generation sequencing technology. Among the differentially expressed genes, a total of 147 genes (Additional file 1: Table S3), 73 upregulated and 74 downregulated genes in BUT3-DE vs. Control (BUT1-DE) met the selection criteria of (1) genes that changed by fragments per kilobase of exon per million fragments mapped reads (FPKM) > 0.3 [60] and (2) a minimum of a twofold change in normalized read counts between groups. The P-value (P < 0.05) was estimated for each gene and was corrected for multiple testing using the Benjamini-Hochberg correction. The fold-change was used to partition the genes into up- and downregulated groups. Then, all the significantly expressed genes were subjected to KEGG (Kyoto Encyclopedia of Genes and Genomes) pathway analysis (database from KEGG *E. coli* str. K12 substr. W3110). We identified five significant KEGG pathways in the BUT3-DE OmpC-TMT-expressing strain, including oxidative phosphorylation (4 genes), fructose and mannose metabolism (3 genes), ribosome (4 genes), glycolysis/gluconeogenesis (2 genes) and nicotinate and nicotinamide metabolism (2 genes). All 15 significantly expressed genes were compared between BUT3-DE and BUT1-DE (Table 4). We observed 4 downregulated genes (*nuoI*, *nuoG*, *nuoF* and *nuoC*) in oxidative phosphorylation (Table 4). The *nuo* operon encodes the NADH ubiquinone oxidoreductase (complex I) that couples the transfer of electrons from NADH to ubiquinone with the translocation of protons across the cytoplasmic membrane and forms an integral part of oxidative phosphorylation [61]. Quinones are electron carriers with an isoprenoid side chain that anchors them to the membrane; they function as the primary electron carriers for respiration and are thought to regulate the ArcA–ArcB two-component system in response to the cellular redox state [62–64].

**Table 4:** KEGG biological pathways for significantly expressed genes ^a^

Groups	Gene	Expression difference^b^	Description
Oxidative phosphorylation	*nuoI*	-1.88 ↓	NADH:ubiquinone oxidoreductase, chain I
	*nuoG*	-1.71 ↓	NADH:ubiquinone oxidoreductase, chain G
	*nuoF*	-1.49 ↓	NADH:ubiquinone oxidoreductase, chain F
	*nuoC*	-1.35 ↓	NADH:ubiquinone oxidoreductase, fused CD subunit
Fructose and mannose metabolism	*fucA*	2.26 ↑	L-fuculose-1-phosphate aldolase
	*srlE*	1.63 ↑	glucitol/sorbitol-specific enzyme IIB component of PTS
	*srlA*	2.78 ↑	glucitol/sorbitol-specific enzyme IIC component of PTS
Ribosome	*rplA*	-1.32 ↓	50S ribosomal subunit protein L1
	*rpsF*	-1.42 ↓	30S ribosomal subunit protein S6
	*rpmF*	-2.22 ↓	50S ribosomal subunit protein L32
	*rplI*	-1.34 ↓	50S ribosomal subunit protein L9
Glycolysis/Gluconeogenesis	*pfkB*	-1.75 ↓	6-phosphofructokinase II
	*pgm*	-1.44 ↓	phosphoglucomutase
Nicotinate and nicotinamide metabolism	*yjjG*	1.24 ↑	dUMP phosphatase
	*pntA*	-1.45 ↓	pyridine nucleotide transhydrogenase, alpha subunit

^**a**^KEGG pathway annotation enrichment analysis for *E. coli str.* K12 substr. W3110 (Org code : ecj)

^**b**^Genes that changed by FPKM > 0.3 and ≧ 2-fold differences between BUT3-DE vs. BUT1-DE

↑Represents up-regulated genes; ↓ represents down-regulated genes

However, the past results showed that isobutanol disrupts the cell membrane, leading to quinone malfunction, which results in the release of quinone inhibition on ArcB and the subsequent autophosphorylation of ArcB and activation of ArcA, which adapts cellular metabolism for growth with decreased respiratory efficiency, such as downregulation of NADH dehydrogenase I (*nuo* operon) or upregulation of cytochrome d oxidase (*cydAB* operon) [16]. This hypothesis coincides with our results. In addition, microbial fermentation pathways involve many redox reactions, which usually require NADH and NAD^+^ as cofactors. However, in the *clostridial* synthetic pathway, the synthesis of n-butanol from glucose can cause an NADH/NAD^+^ redox imbalance because more NADH is required in the synthetic pathway than is generated in the glycolytic pathway [65]. Therefore, compared with the control strain, in the modified strain, a number of upregulated genes, such as *fucA*, *srlE* and *srlA*, may help supplement metabolic intermediates for fructose and mannose metabolism to generate NADH and assist in regulating NADH/NAD^+^ redox [66, 67]. These results suggested that maintaining the redox balance of NADH and NAD^+^ is a key to ensure the continued operation of cellular metabolism under n-butanol fermentation. The same strategy was used in previous research [8, 65]. Moreover, isobutanol disrupts quinone/quinol function, resulting in the malfunction of NADH ubiquinone oxidoreductase (complex I), which would diminish the ability to convert NADH to NAD^+^ and generate the proton motive force (PMF) [16]. This process may affect the biosynthesis of ATP in the cell. We also found that two genes (*pfkB*, *pgm*) involved in glycolysis/gluconeogenesis were downregulated. One possible explanation is that the downregulation of *pfkB*, which encodes 6-phosphofructokinase II, results in the decreased conversion of fructose-6-phosphate (F6P) to fructose-1, 6-biphosphate, thereby reducing the consumption of ATP. Additionally, *pgm* encodes phosphoglucomutase and facilitates the interconversion of glucose-1-phosphate and glucose-6-phosphate (G6P). One possible explanation for the downregulation of *pgm* is that glucose-1-phosphate is further converted to ADP-glucose by ATP depletion. This result indicated that BUT3-DE tried to reduce ATP consumption following NADH dehydrogenase I malfunction. Moreover, both the *pfkB* and *pgm* genes were downregulated, which may result in the accumulation of G6P and F6P. Both G6P and F6P, as starting metabolites for the pentose phosphate pathway, produce precursors and provide a major source of NADPH for biosynthesis, which is involved in most of reductive pathways [68, 69]. However, previous studies show the energy-dependent reduction of NADP^+^ with NADH by PntAB, but increased NADPH formation will downregulate *pntA* for genetic and environmental manipulation [70].

In general, cells exposed to n-butanol show elevated levels of oxidative stress, and many oxidative-related genes have been identified as being significantly upregulated [17]. However, in our case, although BUT3-DE showed elevated butanol production and higher radical scavenging capacity than BUT1-DE (Figs. 3b and 5b), we did not detect significant expression of oxidative stress-related genes. In fact, our analysis found that genes related to oxidative stress (*sodA*) and cell envelope stress (*cpxP*) were expressed (Additional file 1: Table S3). Meanwhile, KEGG pathway analysis revealed no significant differences between BUT1-DE and BUT3-DE with respect to these genes, and thus was not included in Table 4. Given that BUT1-DE experienced higher oxidative stress than BUT3-DE (Fig. 5b), these results suggest that OmpC-TMT overexpression decreased ROS production, as well as the expression of oxidative stress-related genes, in BUT3-DE.

## Conclusions

This study uses a novel approach to develop *E. coli* strains that express both the n-butanol synthesis pathway and membrane-targeted MTs to improve cell fermentation via ROS scavenging. OmpC-TMT was able to decrease the production of free radicals and to improve host robustness during fermentation. Furthermore, a balanced intracellular redox state in microbes is recognized as essential to ensure the efficient production of fermentation products. Unfortunately, our studies suggest that although OmpC-TMT expression increased n-butanol production compared to that of the control strain, the accumulation of fermented products (e.g., n-butanol) in the cell or external environment cause malfunctions in the NADH and ATP synthesis system. Therefore, this fermentation system requires further improvement. In principle, the expression of membrane-targeted TMT has the potential to improve the alcohol fermentation bioprocess and may also serve as a practical strategy for the construction of platform *E. coli* strains for biofuel production.

## Methods

### Reagents

All chemicals and reagents were purchased from Sigma-Aldrich Co., USA, unless otherwise noted. The reagents, when available, were molecular biology grade. All solutions were prepared using these reagents and sterile distilled water.

### Construction of plasmids via the OGAB method

The OGAB method was described previously [29]. The primer sequences used in this study are listed in Additional file 2: Table S1. To construct an expression plasmid containing the clostridial n-butanol synthesis pathway, *thil* (1.2 kb) and the *crt*-*bcd*-*etfB*-*etfA*-*hbd* cluster (4.8 kb) were first amplified from the genomic DNA of *C. acetobutylicum* ATCC824, and the *adhel* (2.7 kb) gene was amplified from *C. acetobutylicum* ATCC824 megaplasmid pSOL1. *ompC*-*tmt* (1.3 kb) was amplified from pET-OmTmt [27]. The PCR products were subcloned into the yT&A cloning vector (YEASTERN) and confirmed by DNA sequencing. All PCR products were digested and then assembled as one transcriptional unit into pGETS118 in a polycistronic manner using the OGAB method, resulting in pBUT, pBUT-rO-tMT and pBUT-T7-rO-tMT (Fig. 1). All genes and an assembly vector were prepared as DNA fragments with arbitrary 3’-nucleotide protrusions that allowed the genes to be linked together in a specific order and direction. The protrusions were generated by type II restriction endonucleases that recognize two separate recognition sites, such as *SfiI*, which recognizes 5’-GGCCANNNAGGCC-3’, where N is any nucleotide in a random sequence. *SfiI* and *BglI* were chosen in this study since *SfiI* and *BglI* sites are absent in the relevant genes and vectors. The constructed plasmids were transferred into *E. coli* JM109 and JM109 (DE3) to obtain the recombinant *E. coli* strains BUT1, BUT2, BUT3, BUT1-DE, BUT2-DE and BUT3-DE (Table 1) and to assay the production of n-butanol.

### Bacterial strains, culture media and growth conditions

MT-expressing engineered strains, protein expression and their locations in recombinants *E. coli* hosts were confirmed in our previous study [27]. All batch cultures were grown at 37 °C in a rotary shaker at 200 rpm. Batch cultures were grown in 150 mL PYG medium under anaerobic conditions (per liter: 5 g of peptone, 10 g of yeast extract, 60 g of glucose, 5 g of tryptone, 40 mg of K_2_HPO_4_, 19.2 mg of MgSO_4_.7 (H_2_O), 8 mg of CaCl_2_, 40 mg of KH_2_PO_4_, 0.4 g of NaHCO_3_, 80 mg of NaCl and 1.1 mg of FeSO_4_.7 H_2_O) or in M9 (AMRESCO-J863) media. Each engineered *E. coli* strain, including PGETS118, BUT1, BUT2, BUT3, PGETS118-DE, BUT1-DE, BUT2-DE, and BUT3-DE (Table 1), was grown in medium supplemented with 50 μg/mL chloramphenicol. When the culture density reached OD 0.6, isopropyl-β-D-thiogalactopyranoside (IPTG) was added to a final culture concentration of 0.6 mM. After 12 h of incubation, cells were harvested for ROS and n-butanol production experiments. All solvent concentrations in media are reported as mg/L.

### Analytical methods

Cell density was measured at 600 nm using a UV–vis spectrophotometer (GENESYS 10S, Thermo Scientific, USA). Fermentation samples were analyzed by gas chromatography (GC) on an Agilent 7890A equipped with a split/splitless injector, flame ionization detector, and a DP-FFAP capillary column (30 m, 0.32 mm i.d., 0.25 μm film thickness). The temperatures of the injector and detector were both maintained at 225 °C. The temperature profile of the column oven was as follows: (i) initial hold at 50 °C for 4 min, increase to 100 °C at 20 °C/min and hold for 1 min; (ii) increase to 170 °C at 30 °C/min and hold for 2.5 min; and (iii) increase to 220 °C at 20 °C/min and hold for 4 min. Nitrogen gas was used as the carrier gas. Split injection mode was used, with a split ratio of 1:10.

### Reactive oxygen species detected by carboxy-H_2_DCFDA under n-butanol stress

The engineered *E. coli* strains were cultured in PYG medium, and different concentrations of n*-*butanol were produced after 0, 20, 36 and 60 h of incubation. Aliquots of 100 μL of the cultured strains were re-suspended in 5 mL of M9 medium, and 140 μL of each diluted sample was transferred to a 96-well plate, followed by incubation at 37 °C. The assay method was adapted from a previous study [17]. All samples were treated with 10 μL of 25 mM carboxy-H_2_DCFDA (Invitrogen, Co., Carlsbad, CA) and incubated at 37 °C for 15 min. The optical density at 600 nm and the fluorescence excitation/emission at 535/600 nm of each sample were measured on a plate reader. Tert-butyl hydroperoxide (TBHP) (Invitrogen, Carlsbad, CA) is a known stressor that produces intracellular H_2_O_2_; a set of positive controls for the ROS assay were prepared with strains cultured after inoculation and were treated as above, except with an initial dilution step with 10 μL of 7.78 M TBHP.

### Staining of bacterial suspensions with DAPI and SYTOX Green

Bacterial suspensions containing either 1.0 × 10^6^ or 1.2 × 10^6^ organisms/mL were stained with 0.5 μM SYTOX Green and 1 μg/ml DAPI. The fluorescence emissions from *E. coli* were compared with the background fluorescence of each stain alone at the optimal excitation wavelength for each nucleic acid stain (DAPI, 358 nm; SYTOX Green, 504 nm). The mixture of cells and DAPI was incubated at 37 °C for 10 min, and the cells were washed twice with TBS buffer (pH 7.6). Next, the DAPI-stained cells were stained with SYTOX Green nucleic acid again, incubated at 37 °C for 10 min, and washed three times with TBS buffer (pH 7.6). Fluorescence was observed on a Nikon ECLIPSE 80i (Nikon, Tokyo, Japan).

### Next-generation sequencing and analysis

After 60 h of incubation, total RNA was isolated from both butanologenic strains (BUT1-DE and BUT3-DE) for next-generation sequencing (RNA-Seq), which was performed by Welgene Biotech Co., Ltd. (Taipei, Taiwan). Total RNA was extracted using the Trizol® Reagent (Invitrogen, USA) according to the manufacturer’s instructions. Purified RNA was quantified at 260 nm using an ND-1000 spectrophotometer (Nanodrop Technology, USA) and was qualitatively analyzed using a Bioanalyzer 2100 (Agilent Technology, USA) with an RNA 6000 labchip kit (Agilent Technologies, USA). All procedures were performed according to the Illumina protocol. For all samples, library construction was performed using TruSeq RNA Sample Prep Kits v2 for 75 bp (Single-End) sequencing and the Solexa platform (Illumina Inc.). Sequences were directly determined using sequencing-by-synthesis technology via the TruSeq SBS Kit. Raw sequences were obtained from the Illumina Pipeline software bcl2fastq v2.0 and were expected to generate 10 M (million reads or Gb) per sample. The generated sequences were filtered to obtain qualified reads. Trimmomatics was implemented to trim or remove reads according to the quality score [71]. Qualified reads after filtering low-quality data were analyzed using TopHat/Cufflinks for gene expression estimation [72]. The gene expression level was calculated as FPKM (fragments per kilobase of transcript per million mapped reads). For differential expression analysis, CummeRbund was employed to perform statistical analysis of the gene expression profiles. The reference genome and gene annotations were retrieved from the Ensembl database for *E. coli* str. K-12 substr. W3110. The cuffdiff tool from the cufflinks package was run to calculate expression changes and the associated P-values for each gene between strain BUT1-DE (control) and strain BUT3-DE (Table 1) after 60 h of incubation. The output files of cuffdiff were annotated by adding gene functional descriptions. KEGG term enrichment analysis and fold-change enrichment for the gene lists of significantly upregulated and downregulated genes in two butanologenic strains were performed. KEGG pathways were analyzed using NIH DAVID Bioinformatics Resources 6.7 [73] to identify the regulated biological themes.

### Quantitative Real-Time PCR Analysis

Total RNA for quantitative reverse transcriptase PCR (qPCR) was extracted as described for next-generation sequencing and analysis. Two-step quantitative real-time PCR (qPCR) was conducted using LightCycler ® FastStart DNA Master PLUS SYBR Green I (Roche Life Science) on a Light Cycler 480 Real-Time PCR 3System (Roche Co., Germany) and was performed by Welgene Biotech Co., Ltd. (Taipei, Taiwan). The four genetic regions involved in n-butanol production pathway (*thil*, *crt*-*bcd*-*etfB*-*etfA*-*hbd*, *adhe*) and ROS-targeted scavenging (*ompC*-*tmt*) were analyzed. R16s (*rrsA* gene) was used as a reference in all analyses. The primers are provided in Additional file 3: Table S2 in the supplemental material. For the relative quantification of gene expression, the comparative CT method was employed. The averaged CT was subtracted from the corresponding averaged r16S value for each sample; resulting in ∆CT. ∆∆CT was obtained by subtracting the average control ∆CT value from the average experimental ∆CT. The fold increase was determined by calculating log2 (2^−∆∆CT^) for the experimental vs. control samples.

## Additional files

Additional file 1: Table S3. List of the 147 differentially expressed genes. In all 147 differentially expressed genes, including 132 down- and 4 up-regulated, were detected in the BUT3-DE, compared to the BUT1-DE. (DOCX 37 kb)

Additional file 2: Table S1. Primes used for OGAB method. The primer sequences used in this study are listed. (DOCX 23 kb)

Additional file 3: Table S2. Primes used for qPCR. The primer sequences used in this study are listed. (DOCX 22 kb)

## Abbreviations

PYG: Medium containing peptone, yeast extract and glucose.
ROS: Reactive oxygen species
