# Supplementary material for: Improved n-butanol production via co-expression of membrane-targeted tilapia metallothionein and the clostridial metabolic pathway in Escherichia coli
Source: BMC Biotechnol. 2017 Apr 11;17:36. doi: 10.1186/s12896-017-0356-3 (PMC5387206; doi:10.1186/s12896-017-0356-3)
Supplement: Supplementary file 1 — List of the 147 differentially expressed genes. In all 147 differentially expressed genes, including 132 down- and 4 up-regulated, were detected in the BUT3-DE, compared to the BUT1-DE. (DOCX 37 kb) [file 12896_2017_356_MOESM1_ESM.docx]

**Improvement of n-butanol production via co-expression of membrane-targeted tilapia metallothionein and clostridial metabolic pathway in *E. coli***

# Supplementary Online Material

# Additional file 1. List of the 147 differentially expressed genes (Table S3)

**Table S3** List of the 147 differentially expressed genes

| Gene ID | Gene Symbol | log2 fold change | Statistics |
| --- | --- | --- | --- |
|  |  | BUT3-DE / BUT1-DE | P value |
| BAE77586 | *tnaC* | 24.31 | 2.80E-02 |
| EBG00000041726 | *selC* | 22.95 | 9.00E-03 |
| BAE76455 | *yneL* | 21.98 | 5.00E-05 |
| BAE76923 | *ygeK* | 18.35 | 5.00E-05 |
| BAE76449 | *yddJ* | 16.82 | 9.00E-03 |
| BAA35294 | *ybeT* | 16.68 | 2.05E-03 |
| BAA15998 | *ompC* | 3.06 | 9.25E-03 |
| BAA18886 | *srlA* | 2.78 | 3.03E-02 |
| BAE76549 | *yecT* | 2.63 | 3.02E-02 |
| BAE76872 | *fucA* | 2.26 | 3.58E-02 |
| BAE76926 | *ygeO* | 2.14 | 4.49E-02 |
| BAA16159 | *cvpA* | 2.14 | 4.11E-02 |
| BAE77143 | *exuT* | 2.06 | 6.00E-03 |
| BAA15957 | *gatA* | 1.89 | 9.50E-03 |
| BAA15204 | *uxaB* | 1.87 | 1.34E-02 |
| BAE77921 | *yhfL* | 1.87 | 4.05E-03 |
| BAA15082 | *yncC* | 1.68 | 2.03E-02 |
| BAA16564 | *srlE* | 1.63 | 2.31E-02 |
| BAE77759 | *bcsE* | 1.62 | 2.85E-02 |
| BAE77565 | *bglG* | 1.60 | 2.55E-02 |
| BAE77393 | *sbp* | 1.60 | 1.42E-02 |
| BAE78205 | *yjfZ* | 1.57 | 1.49E-02 |
| BAE78216 | *ytfI* | 1.56 | 1.05E-02 |
| BAA35356 | *kdpA* | 1.56 | 1.11E-02 |
| BAE76522 | *ynjF* | 1.54 | 1.67E-02 |
| BAE77174 | *garP* | 1.54 | 2.48E-02 |
| BAE78085 | *yjcS* | 1.54 | 1.03E-02 |
| BAE77166 | *tdcB* | 1.52 | 2.85E-02 |

**Table S4 Cont.**

| Gene ID | Gene Symbol | log2 fold change | Statistics |
| --- | --- | --- | --- |
|  |  | BUT3-DE / BUT1-DE | P value |
| BAE77033 | *pppA* | 1.50 | 2.42E-02 |
| BAE77635 | *setC* | 1.50 | 2.58E-02 |
| BAE76621 | *sanA* | 1.47 | 1.57E-02 |
| BAE77571 | *cbrC* | 1.47 | 3.95E-02 |
| BAE78091 | *alsB* | 1.45 | 2.40E-02 |
| BAE78183 | *yjfJ* | 1.43 | 1.91E-02 |
| BAE77167 | *tdcA* | 1.42 | 2.57E-02 |
| EBG00000041682 | *rrsC* | 1.42 | 1.91E-02 |
| BAA15223 | *eamA* | 1.42 | 1.79E-02 |
| BAA15444 | *ydhY* | 1.40 | 2.69E-02 |
| BAE77446 | *yihG* | 1.40 | 2.99E-02 |
| BAA15224 | *ydeE* | 1.40 | 2.29E-02 |
| BAA16372 | *hyfD* | 1.39 | 4.68E-02 |
| BAE77809 | *yhhI* | 1.38 | 4.18E-02 |
| BAE78356 | *bglJ* | 1.38 | 2.15E-02 |
| BAA15726 | *yecI* | 1.38 | 2.13E-02 |
| BAE77396 | *cpxP* | 1.38 | 3.02E-02 |
| BAE77480 | *pldA* | 1.37 | 2.17E-02 |
| BAE76280 | *ylbG* | 1.35 | 2.13E-02 |
| BAE76281 | *ybbB* | 1.34 | 2.33E-02 |
| BAE77620 | *emrD* | 1.34 | 2.30E-02 |
| BAE76811 | *pphB* | 1.33 | 3.06E-02 |
| BAE78160 | *yjeM* | 1.32 | 2.68E-02 |
| BAE76485 | *ydgK* | 1.31 | 4.28E-02 |
| BAE78181 | *rlmB* | 1.30 | 4.08E-02 |
| BAE78318 | *yjiC* | 1.30 | 3.93E-02 |
| BAE76195 | *ribE* | 1.30 | 3.41E-02 |
| BAA16576 | *yqiH* | 1.28 | 4.98E-02 |
| BAE76524 | *ynjI* | 1.28 | 2.86E-02 |
| BAE78309 | *fimC* | 1.26 | 3.85E-02 |
| BAA15560 | *ydjE* | 1.26 | 4.09E-02 |
| BAE78362 | *yjjG* | 1.24 | 3.56E-02 |
| BAA15964 | *gatA* | 1.23 | 4.23E-02 |

**Table S4 Cont.**

| Gene ID | Gene Symbol | log2 fold change | Statistics |
| --- | --- | --- | --- |
|  |  | BUT3-DE / BUT1-DE | P value |
| BAB96645 | *leuO* | 1.23 | 3.76E-02 |
| BAA14776 | *oppB* | 1.23 | 4.06E-02 |
| BAA35700 | *pyrD* | 1.23 | 4.00E-02 |
| BAE76599 | *yehR* | 1.21 | 4.58E-02 |
| EBG00000041657 | *rrsE* | 1.20 | 4.20E-02 |
| BAA14929 | *abgT* | 1.20 | 4.12E-02 |
| BAE76157 | *ampH* | 1.20 | 4.08E-02 |
| BAE77401 | *sodA* | 1.20 | 4.20E-02 |
| BAE78308 | *fimI* | 1.19 | 4.75E-02 |
| BAE78289 | *yjhH* | 1.19 | 4.25E-02 |
| BAE78316 | *uxuB* | 1.18 | 4.19E-02 |
| BAE76910 | *araE* | 1.13 | 4.78E-02 |
| BAE76494 | *gloA* | -1.14 | 4.77E-02 |
| BAA35270 | *tatE* | -1.15 | 4.98E-02 |
| BAA35406 | *tolB* | -1.17 | 4.32E-02 |
| BAA15731 | *yecA* | -1.20 | 4.02E-02 |
| BAE76303 | *cysS* | -1.22 | 3.59E-02 |
| BAA35900 | *fabD* | -1.23 | 4.32E-02 |
| BAE78257 | *pepA* | -1.25 | 3.98E-02 |
| BAE77196 | *yraP* | -1.25 | 4.89E-02 |
| BAA15735 | *yecF* | -1.26 | 3.24E-02 |
| BAE78242 | *yjgF* | -1.27 | 3.27E-02 |
| BAA35743 | *appC* | -1.27 | 3.04E-02 |
| BAA36012 | *ycgK* | -1.28 | 3.58E-02 |
| BAE76722 | *ypfH* | -1.28 | 4.04E-02 |
| BAA16497 | *yfjD* | -1.29 | 4.42E-02 |
| BAA35374 | *ybgI* | -1.29 | 2.89E-02 |
| BAE76966 | *bglA* | -1.31 | 3.49E-02 |
| BAE76480 | *ydgD* | -1.32 | 4.26E-02 |
| BAE77336 | *rplA* | -1.32 | 4.87E-02 |
| BAE78204 | *rplI* | -1.34 | 2.46E-02 |
| BAA35835 | *ymdB* | -1.34 | 2.25E-02 |
| BAA16115 | *nuoC* | -1.35 | 2.33E-02 |

**Table S4 Cont.**

| Gene ID | Gene Symbol | log2 fold change | Statistics |
| --- | --- | --- | --- |
|  |  | BUT3-DE / BUT1-DE | P value |
| BAA15634 | *cspC* | -1.38 | 2.11E-02 |
| BAE78113 | *proP* | -1.40 | 3.36E-02 |
| BAE76206 | *yajQ* | -1.42 | 3.09E-02 |
| BAE78201 | *rpsF* | -1.42 | 2.16E-02 |
| BAA35407 | *pal* | -1.43 | 1.60E-02 |
| BAE76819 | *nlpD* | -1.44 | 2.87E-02 |
| BAA35337 | *pgm* | -1.44 | 2.08E-02 |
| BAA15342 | *pntA* | -1.45 | 1.90E-02 |
| BAA15402 | *slyB* | -1.46 | 4.28E-02 |
| BAA35516 | *iaaA* | -1.46 | 1.42E-02 |
| BAA35857 | *yceP* | -1.48 | 3.31E-02 |
| BAA16113 | *nuoF* | -1.49 | 3.84E-02 |
| BAA35584 | *ltaE* | -1.49 | 1.22E-02 |
| BAE78146 | *yjeI* | -1.51 | 1.94E-02 |
| BAE76442 | *ydcY* | -1.52 | 1.21E-02 |
| BAA35875 | *yceH* | -1.52 | 1.42E-02 |
| BAE76495 | *ydhR* | -1.52 | 1.47E-02 |
| BAA35501 | *ybiT* | -1.54 | 1.04E-02 |
| BAA35472 | *ybiM* | -1.55 | 2.98E-02 |
| BAE78241 | *mgtA* | -1.56 | 4.04E-02 |
| BAA36142 | *cbpA* | -1.56 | 2.73E-02 |
| BAA35199 | *ompT* | -1.57 | 2.89E-02 |
| BAE76264 | *ybaS* | -1.63 | 6.75E-03 |
| BAE77783 | *hdeD* | -1.66 | 1.11E-02 |
| BAE76818 | *rpoS* | -1.68 | 1.13E-02 |
| BAA16111 | *nuoG* | -1.71 | 1.17E-02 |
| BAE76798 | *hycE* | -1.72 | 4.15E-03 |
| BAA15500 | *pfkB* | -1.75 | 4.10E-03 |
| BAA15556 | *ydjA* | -1.76 | 1.78E-02 |
| BAA15728 | *ftn* | -1.82 | 3.85E-03 |
| BAE77159 | *yhaO* | -1.86 | 3.45E-03 |
| BAA16109 | *nuoI* | -1.88 | 1.23E-02 |
| BAA35467 | *ybiC* | -1.90 | 1.85E-03 |

**Table S4 Cont.**

| Gene ID | Gene Symbol | log2 fold change | Statistics |
| --- | --- | --- | --- |
|  |  | BUT3-DE / BUT1-DE | P value |
| BAE77151 | *yqjF* | -1.96 | 2.45E-03 |
| BAA16044 | *lpp* | -1.98 | 1.27E-02 |
| BAA35896 | *yceD* | -1.98 | 1.65E-03 |
| BAA15983 | *spr* | -2.04 | 9.20E-03 |
| BAE77095 | *zupT* | -2.12 | 1.00E-03 |
| BAA35897 | *rpmF* | -2.22 | 4.50E-04 |
| BAE77785 | *hdeB* | -2.22 | 2.30E-03 |
| BAE76799 | *hycD* | -2.25 | 3.60E-03 |
| BAE77784 | *hdeA* | -2.40 | 1.90E-03 |
| BAA15331 | *asr* | -2.51 | 4.15E-03 |
| BAE77157 | *yhaL* | -2.63 | 2.05E-02 |
| BAE77854 | *yhhW* | -3.30 | 5.00E-05 |
| BAE76432 | *ynbD* | -3.33 | 5.00E-05 |
| BAE77158 | *yhaM* | -3.60 | 5.00E-05 |
| BAA35468 | *ybiJ* | -4.50 | 3.50E-04 |
| BAA15024 | *azoR* | -4.57 | 5.00E-05 |
| BAE77156 | *yhaK* | -5.59 | 5.00E-05 |
| BAE77738 | *hokA* | -19.69 | 4.82E-02 |
| EBG00000041721 | *valW* | -23.64 | 4.82E-02 |
| BAE76026 | *thrL* | -25.42 | 4.82E-02 |

Genes that changed by FPKM > 0.3 and ≧ 2-fold differences between BUT3-DE vs. BUT1-DE. Genes are sorted by fold changes in descending order.
