# Supplementary material for: Improved n-butanol production via co-expression of membrane-targeted tilapia metallothionein and the clostridial metabolic pathway in Escherichia coli
Source: BMC Biotechnol. 2017 Apr 11;17:36. doi: 10.1186/s12896-017-0356-3 (PMC5387206; doi:10.1186/s12896-017-0356-3)
Supplement: Supplementary file 2 — Primes used for OGAB method. The primer sequences used in this study are listed. (DOCX 23 kb) [file 12896_2017_356_MOESM2_ESM.docx]

**Improvement of n-butanol production via co-expression of membrane-targeted tilapia metallothionein and clostridial metabolic pathway in *E. coli***

# Supplementary Online Material

# Additional file 2. Primes used for OGAB method (Table S1)

**Table S1** Primes used for OGAB method ^a^

| Oligonucleotide | Target gene | Sequence (5’-3’) | Separate recognition sites |
| --- | --- | --- | --- |
| Primer 1 - F | *thil* | TAGGGCCA**GTT**AGGCCTATTATTTAGGAGGAGTAAAACATGAGAGATGTAG | SfiI (GTT) |
| Primer 2 - R |  | TAGGGCCA**TAG**AGGCCTTAGTCTCTTTCAACTACGAGAGC | SfiI (CTA) |
| Primer 3 - F | *crt*-*bcd*-*etfAB*-*hbd* | TAGGCCA**CTA**AGGCTATTTTAGGAGGATTAGTCATGGAACTAAAC | BglI (CTA) |
| Primer 4 - R |  | TAGGCCA**CAC**AGGCTTATTTTGAATAATCGTAGAAACCTTTTCCTGATTTTC | BglI (GTG) |
| Primer 5 - F | *adhe* | TAGGCCA**GTG**AGGCTTATAAAGGAGTGTATATAAATGAAAGTTACAAATCAAAAAGAAC | BglI (GTG) |
| Primer 6 - R |  | TAGGCCA**AGA**AGGCTTAAAATGAT TTTATATAGA TATCCTTAAG TTCACTTATA AGTGG | BglI (TCT) |
| Primer 7 - F | rbs-*ompC-tmt* | TAGGGCCA**ACA**AGGCCTAACTTTAAG AAGGAGATAT ACATATGAAA GTTAAAGTAC | SfiI (ACA) |
| Primer 8 - R |  | TAGGGCCA**AGA**AGGCCTTAGAACTGGTAAACCAGACCCAGAGCTACGATGTTATCA | SfiI (TCT) |
| Primer 9 - F | T7 promoter-*ompC-tmt* | TAGGGCCA**ACA**AGGCCTAATACGACTCACTATAGGGGAATTGTGAGCGGATAAC | SfiI (ACA) |
| Primer 10 - R |  | TAGGGCCA**AGA**AGGCCTTAGAACTGGTAAACCAGACCCAGAGCTACGATGTTATCA | SfiI (TCT) |

^a^ Underlined nucleotides are type II restriction endonucleases recognition sites, while bold letters nucleotides indicate protrusions generated by type II restriction enzyme digestion.
