# Supplementary material for: Improved n-butanol production via co-expression of membrane-targeted tilapia metallothionein and the clostridial metabolic pathway in Escherichia coli
Source: BMC Biotechnol. 2017 Apr 11;17:36. doi: 10.1186/s12896-017-0356-3 (PMC5387206; doi:10.1186/s12896-017-0356-3)
Supplement: Supplementary file 3 — Primes used for qPCR. The primer sequences used in this study are listed. (DOCX 22 kb) [file 12896_2017_356_MOESM3_ESM.docx]

**Improvement of n-butanol production via co-expression of membrane-targeted tilapia metallothionein and clostridial metabolic pathway in *E. coli***

# Supplementary Online Material

# Additional file 3. Primes used for qPCR (Table S2)

**Table S2** Primers used for qPCR

| Oligonucleotide | Target gene | Sequence (5’-3’) | PCR product size |
| --- | --- | --- | --- |
| Primer 1 - F | *thil* | CCAGCAAGACAAGCAGCAG | 145 |
| Primer 2 - R |  | CTACTACAATGGTATCAGCATCTCC | 145 |
| Primer 3 - F | *crt*-*bcd*-*etfAB*-*hbd* | TTAGGAGGCGGATGCGAAATAG | 145 |
| Primer 4 - R |  | TGCCCATTCCAACTAATCTTGAAAG | 145 |
| Primer 5 - F | *adhe* | TCGGTTATGGCTACGGATTATACTG | 114 |
| Primer 6 - R |  | TTCTCTTGCTTCAATGTCGTTAGTC | 114 |
| Primer 7 - F | T7 promoter-*ompC-tmt* | CCAGACCTACAACGCAACTCG | 108 |
| Primer 8 - R |  | AGGGACGGACGCAGACC | 108 |
| Primer 9 - F | 16S | TGGAGGAAGGTGGGGATGAC | 131 |
| Primer 10 - R |  | GGACTACGACGCACTTTATGAGG | 131 |
